# A Rare Case of Left Hemicolectomy Masking the Signs and Symptoms of Underlying Hirschsprung’s Disease in an Adult

**DOI:** 10.7759/cureus.46533

**Published:** 2023-10-05

**Authors:** Creighton Kellogg, Lori A Robbins

**Affiliations:** 1 Medical School, Carolina Campus, Edward Via College of Osteopathic Medicine, Spartanburg, USA; 2 Gastroenterology, Palmetto Digestive Disease and Endoscopy Center, Bon Secours Roper St. Francis Hospital, Charleston, USA

**Keywords:** colon, constipation, rectoanal inhibitory reflex, left hemicolectomy, hirschsprung's disease

## Abstract

Hirschsprung’s disease (HD) is a congenital gastrointestinal condition characterized by the lack of ganglion cells within the submucosal and myenteric nervous plexuses in the large intestine. This results in a dysfunctional segment of the large colon, resulting in symptoms such as failure to pass meconium, constipation, and dilated loops of the bowel. The vast majority of patients are diagnosed during the neonatal period, but a handful can be diagnosed later into childhood and adolescence. A rare subset is diagnosed during adulthood, in which the section of the aganglionic colon is minimal yet symptomatic. We report the case of a 54-year-old female presenting with dilated loops of bowel and a remote history of severe constipation, recurrent bowel obstructions, previous left hemicolectomy, and an improvement of symptoms following the procedure. Upon further workup, she was diagnosed with HD, raising the question of whether there should be increased testing for this condition in adults. This case can serve as an example of the need for a more in-depth workup of severe constipation in adults, as the finding for HD in adults is rare but still possible.

## Introduction

Hirschsprung’s disease (HD) is a congenital disease that involves the failure of neural crest cells to migrate to the colon during fetal development. This results in an absence of the enteric nervous system in a variable length of the distal colon [1]. The neural crest cells derive from the neural fold and migrate once the neural tube is formed during embryogenesis. Neural crest cells specific for the enteric nervous system migrate along the embryonic gut mesenchyme and eventually help form ganglia within the distal colon. In HD, the neural crest cells can fail to proliferate, migrate, differentiate, and/or survive, resulting in aganglionosis of a certain length of the large bowel. HD is typically confined to the rectum (79.8% of cases), but some patients can have extension into the sigmoid colon (12.5%) and descending colon (0.8%) [2]. These lengths can also be used to specify the type of HD, as there is “long segment” HD with involvement of ascending colon, “short segment” HD with involvement of rectum and sigmoid colon, and then “ultra-short segment” HD with missing ganglion cells in just the rectum.

HD has a prevalence of about one in every 5,000 births, with an overall male predominance of 3:1 to 4:1 [3]. The majority of patients with HD are diagnosed during the neonatal period upon symptoms of distal intestinal obstruction. These can include failure to pass meconium, bilious emesis, and abdominal distension [4]. However, passage of meconium does not exclude the diagnosis of HD, as neonates can develop other obstructive symptoms a few days after birth. Another initial presentation of HD is enterocolitis, which presents with fever, abdominal distension, and foul-smelling stools [5]. HD-associated enterocolitis is a leading cause of mortality in those who develop HD.

The diagnosis of HD involves a combination of imaging and biopsy. The first step in the diagnosis of a neonate with intestinal obstruction symptoms is an abdominal radiograph to assess for obstruction and dilated bowel. A more specific test for HD involves an unprepped contrast enema as well as a rectal suction biopsy. Anorectal manometry is another diagnostic option for patients older than one month. A contrast enema is useful in delineating the transition zone, which is the area between the normal colon and the aganglionic dilated colon. It is also useful for surgeons to know which sections of the bowel to remove [6]. Discovering a clear transition zone is pathognomonic for HD, but the gold standard for diagnosis of HD is rectal suction biopsy [7]. A rectal suction biopsy involves taking a tissue sample of the distal colon at least two centimeters above the dentate line to avoid the hypoganglionic section of the bowel that is normally present. Another biopsy is taken proximal to the original biopsy. If ganglion cells are absent from the biopsies, then this is diagnostic of HD.

With the prevalence of HD being one in every 5,000 births, only a few cases are diagnosed beyond the first few months of life. A majority of these outliers are diagnosed before the age of five, but a rare subset of these is classified as “Adult Hirschsprung’s Disease” with age of onset after 10 years old [8]. The majority of these patients are diagnosed in their 20s and early 30s. More specifically, these patients typically suffer from ultra-short-segment HD with missing ganglion cells in just the rectum only, which can potentially explain how these adults are diagnosed years after birth. The most common clinical presentations involve chronic constipation, intestinal obstruction, fecal impaction, and failure of laxatives and other home remedies. Most of these patients move on to surgical treatment, with a few different procedure options.

## Case presentation

A 54-year-old African American female presented to the gastroenterology clinic due to an incidental finding of significant dilation of her colon on CT imaging. The patient was planning on undergoing elective breast reduction surgery and was found to be dyspneic on exertion during her pre-operative workup, necessitating a CT. Her past medical history involved episodes of severe constipation and recurrent bowel obstructions during her 20s. She denied a history of delayed passage of meconium as a neonate. The constipation during her adolescent years was managed with laxatives, but she could not recall the type of laxatives used and admitted that these did not effectively treat her constipation. She also mentioned that she tried many different elimination diets with no significant benefit. Her daily exercise involved walking, but was recently unable to perform this due to dyspnea on exertion. She was unaware of any family history significant for gastrointestinal issues. She was previously hospitalized three times for symptoms, which only resulted in treatment with laxatives and lactulose fiber. Each of these hospitalizations involved a workup of a potential bowel obstruction, which was negative for true obstructions. She also suffered episodes of severe constipation, mentioning how she would go up to two weeks without a bowel movement with no improvement with laxatives. At age 36, she underwent a left hemicolectomy procedure due to her recurrent symptoms. This surgery involved the removal of the descending colon and the splenic flexure of the colon, followed by connecting the transverse colon to the sigmoid colon. The removed portions of the colon did not undergo histopathological examination. She did not receive a colostomy at the time of the procedure. Since this procedure, the patient has suffered from minimal bowel symptoms and has daily bowel movements. Her last colonoscopy was in 2018, which was unremarkable. The initial workup involved devoted imaging of her colon as well as colonoscopy.

**Figure 1:**
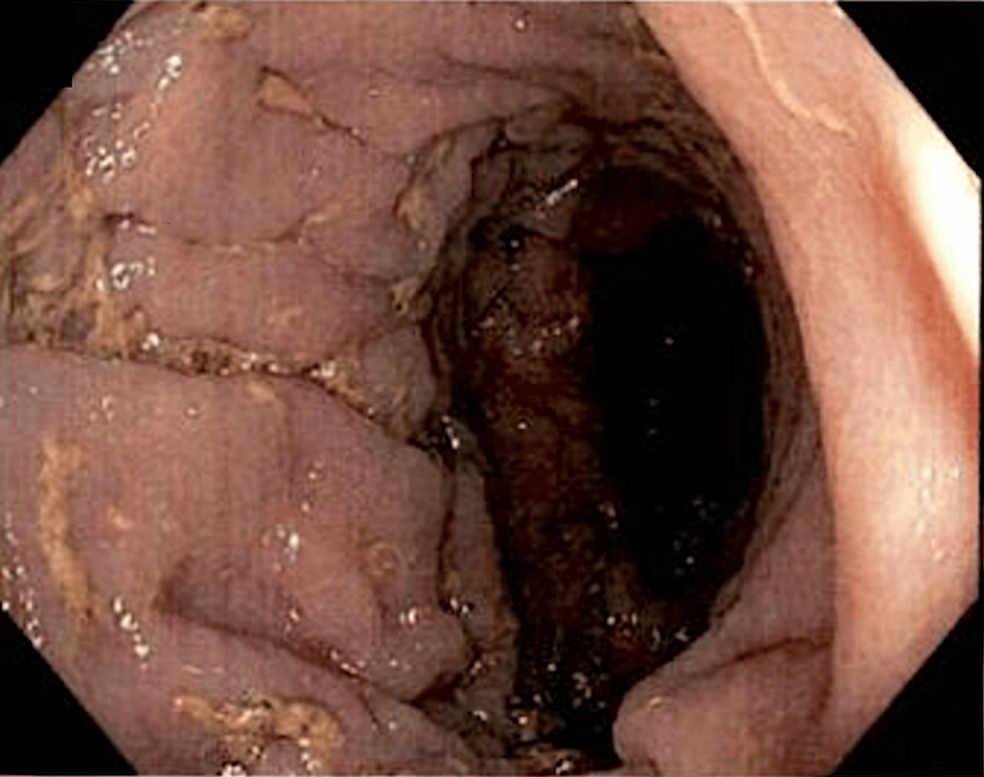
Dilation of the distal transverse colon

**Figure 2:**
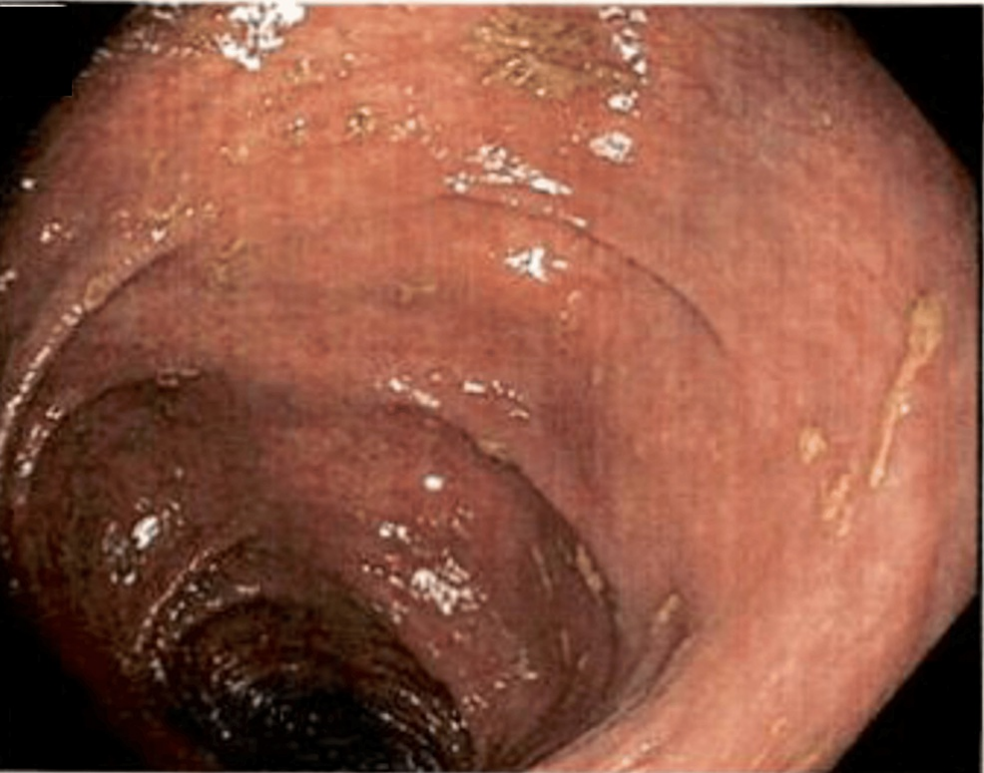
Dilation of the descending colon

**Figure 3:**
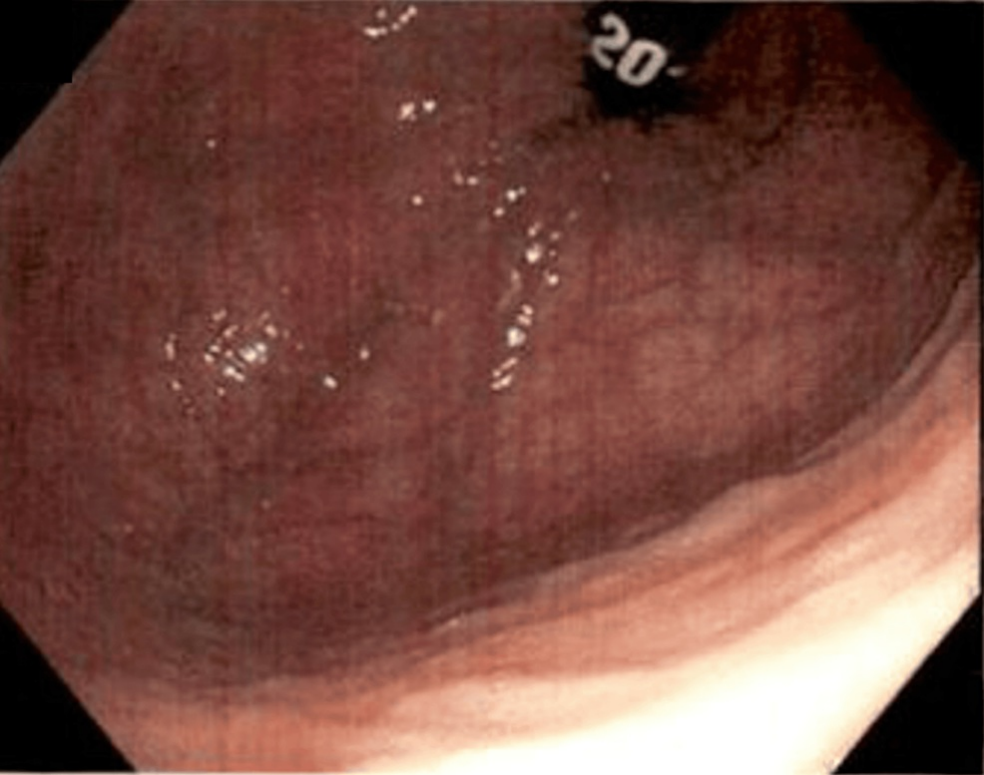
No volvulus in this area distal to the descending colon dilation seen in Figure 2.

Initial imaging involved CT of the abdomen and pelvis with contrast. This showed marked distension of the colon in the proximal descending region, along with abrupt tapering in the distal descending/proximal sigmoid region, after which the colon was completely depressed. There was no obvious mass at the transition point, but the appearance resembled a stricture or internal hernia. The next step involved a barium enema of the lower gastrointestinal (GI) tract. This showed a dilated patulous portion of the colon immediately distal to the anastomotic staple line from her previous left hemicolectomy procedure. This dilated portion was inferior to the asymmetric elevation of the left hemidiaphragm. These findings were consistent on colonoscopy, which showed two areas of marked dilated colon in the distal transverse colon (Figure 1) and the descending colon (Figure 2). There was a minimal twisting of the colon distal to these areas, ruling out sigmoid volvulus as the etiology of these dilations (Figure 3).

The next step involved anorectal manometry due to the unknown etiology of her dilated loops of bowel (Table 1). Anorectal manometry showed normal basal sphincter pressure and squeeze pressure, but there was an absent recto-anal inhibitory reflex (RAIR). The patient was also unable to sense the first sensation sensory threshold, the urge to defecate sensory threshold, and the maximum tolerable sensory threshold. Due to the absence of RAIR, this is suggestive of HD. Due to her history of left hemicolectomy and current asymptomatic state with no bowel complaints, she was instructed to follow up with colorectal surgery, which determined that surgery was not necessary at this time.

**Table 1:** Anorectal Manometry. This patient showed an absent recto-anal inhibitory reflex (RAIR), suggesting a diagnosis of Hirschsprung’s disease. She also showed absent rectal sensations.

Inspection				
Digital Exam	X Able to follow commands on digital exam		[] Unable to follow commands	
	Patient (Actual):		Normal Range:	
Basal Sphincter Pressure (mmHg)	64		50-120 mmHg	
Squeeze Pressure	63		Equal to resting pressure	
RAIR	0 cc		>20 cc	
Strain Maneuver	100%		% relaxation	
Rectal Sensation				
	Patient (Actual):		Normal Range:	
First Sensation	-		40-60 cc	
First Urge	-		80-120 cc	
Maximum Tolerable Volume	-		>200 cc	
Evaluation				
Resting pressure (internal anal sphincter) tone		[] Low	X Normal	[] High
Voluntary muscle (external anal sphincter) contraction		[] Weak	X Normal	[] Strong
Rectal sensation volumes are within the normal range		[] Yes	X No	

## Discussion

With our patient, the absence of the RAIR was clinically sufficient information to allow for a diagnosis of HD. The RAIR is an anal reflex triggered by distension of the rectum by gas or feces, which leads to a relaxation of the internal anal sphincter mediated by the intramural neuronal plexus. During anorectal manometry testing, the RAIR is tested for via inflation of a rectal balloon. The absence of the RAIR is suggestive of a lack of ganglion cells, resulting in colonic dysmotility and subsequent severe constipation [9]. According to a study conducted at Erciyes University in Turkey, the absence of RAIR is diagnostic of HD due to the study representing the lack of internal anal sphincter relaxation in response to rectal distension [10]. This explains the pathophysiology of HD due to a lack of ganglion cells. As a result, this finding is pathognomonic for HD.

The one aspect of this patient that makes this case intriguing is how the diagnosis of HD was made following a colectomy procedure that occurred 18 years ago. Most of her symptoms were before the procedure, and the severity declined following the surgery. With adult-onset HD, the amount of colon that is aganglionic is less when compared to those who become symptomatic during infancy and are diagnosed at a young age. Another term for adult-onset HD is “short-segment” or “ultra-short” segment aganglionosis, based on how much of the colon is aganglionic [11]. With our patient, since her condition was not discovered until adulthood, this indicates she most likely had a variant of short-segment or ultra-short-segment disease. With her left hemicolectomy performed 18 years ago, the segment of the bowel with no ganglion cells could have been taken out, helping to alleviate her symptoms over the next few decades. This remains unknown given her diagnosis was made post-colectomy.

The major significance of this case is due to the late age of diagnosis in our patient. The majority of adult patients who are diagnosed with HD occur in their 20s, with some being found in their 30s. On very rare occasions, a patient is found to have HD beyond the age of 40. The first case of adult HD occurred in 1950 with a man at the age of 54 [12]. Over the last 70 years, only a handful of cases within the literature have described HD being diagnosed in middle-aged adulthood and even in the elderly [13]. With our patient being diagnosed at age 54, this serves as a reminder that HD should be included in the differential diagnosis of adults suffering from treatment-resistant severe constipation without any signs of intestinal obstruction.

As for this patient currently, since she has already undergone a left hemicolectomy and has remained asymptomatic with regular bowel movements, there is no current plan for further surgery.

## Conclusions

Based on the outcome of this patient, the question that should be considered is whether testing for HD should be done more often in young adults who have symptoms of severe constipation and recurrent bowel obstructions. Even though the prevalence of newly diagnosed HD is extremely low in adults, this disease should always be included in the differential diagnosis in the setting of severe constipation and a history of multiple bowel obstructions. In adults who suffer from severe constipation that is refractory to lifestyle changes, laxatives, and medical treatment directed at constipation, the diagnosis of HD should be included in the differential diagnosis. During a colonoscopy in the workup for constipation, a rectal suction biopsy should be taken at the same time to look for ganglion cells. If a patient is presenting the same as the case described here, during a colectomy procedure, the specimen should be sent for histopathological evaluation. One aspect of this patient’s care that could have improved is including HD in the original differential diagnosis during her 20s when her symptoms were the most severe. This would have allowed for more specific testing such as rectal biopsy and anorectal manometry, leading to an earlier diagnosis of HD, and a more precise surgical treatment plan. In adults with severe constipation refractory to treatment, a rectal suction biopsy or anorectal manometry testing should be done to look for a possible diagnosis of HD.
